# The Application of the Biolog EcoPlate Approach in Ecotoxicological Evaluation of Dairy Sewage Sludge

**DOI:** 10.1007/s12010-014-1131-8

**Published:** 2014-08-14

**Authors:** Agata Gryta, Magdalena Frąc, Karolina Oszust

**Affiliations:** Department of Soil and Plant System, Institute of Agrophysics Polish Academy of Sciences, Doswiadczalna 4, 20-290 Lublin, Poland

**Keywords:** Waste, Activated sludge, Ecotoxicity, Metabolic fingerprinting, Microorganism activity

## Abstract

An increasing amount of sewage sludge requires reasonable management, whereas its storage might be environmentally hazardous. Due to the organic matter and nutrient presence in sediments, it may be used as organic fertilizer. However, beyond the valuable contests, sewage sludge can also contain toxic or dangerous ingredients like heavy metals. Therefore, there is a need to develop methods for rapid assessment of sediment ecotoxicity that will determine its possible applicability in agriculture. The Biolog® EcoPlate enables the metabolic profile diversity evaluation of microbial populations in environmental samples, which reflects the state of their activity. It is regarded as a modern technology that by means of biological properties allows quick characterization of the ecological status of environmental samples, such as sewage sludge.

## Introduction

In recent years, an intensively occurring process of urbanization and economic development is observed. It gives a reason to find novel solutions on to address the new ecological and biological issue related to the increasing amount of sewage sludge and their application or utilization [1]. Communal and industrial sewage treatment plants continually produce the increasing amount of sludge [2]. Therefore, the storage of sewage sludge is a threat to the environment, and its efficient use in various areas is essential. The storage of sewage sludge carries the risk of the nutrient penetration into groundwater and consequently leads to its contamination [3–5].

There is an increasing interest in disposal of sewage sludge as organic fertilizers noted. Sewage sludge is the insoluble residue from wastewater treatment, and in general terms, it is very rich in valuable nutrients (Table 1). The chemical composition of sludge depends on the type of depuration treatment and origin of the wastewater. Generally, sewage sludge comprises nitrogen (3 %), phosphorus (2 %), other macronutrients (potassium 0.5 %, calcium 1.5 %), organic matter, organic micropollutants, and microorganisms. The great amount of nitrogen and phosphorus concentration in sludge provides the ability to be useful as fertilizer material, and its organic constituents give beneficial soil-conditioning properties [6]. On the other hand, sewage sludge could contain high concentrations of potentially toxic elements such as heavy metals: zinc, nickel, cadmium, cooper, and lead. Heavy metal accumulation in the plant tissues may also occur. It is obvious that positive effect of sludge addition should be considered as well as potential risk of contamination soil of heavy metals.

**Table 1:** Characteristics of the waste-activated sludge used in the experiment [27]

Parameter	Waste-activated sludge
pH	7.23
Dry matter of sludge (g kg^−1^)	121.3
Corg (g kg^−1^ dwt)	868.0
Ntot (g kg^−1^ dwt)	54.4
Ptot (g kg^−1^ dwt)	33.0
Ktot (g kg^−1^ dwt)	15.6
Zn (mg kg^−1^ dwt)	194.0
Cd (mg kg^−1^ dwt)	0.0
Cu (mg kg^−1^ dwt)	18.7
Pb (mg kg^−1^ dwt)	5.3
Ni (mg kg^−1^ dwt)	21.7
Cr (mg kg^−1^ dwt)	14.1
Hg (mg kg^−1^ dwt)	0.0

The problem of sludge disposal is a huge challenge for science and agriculture whose task is to develop environmentally safe method of their utilization and conducting environmental monitoring during their use. Biological properties of sludge may be used as ecotoxicity indicators.

The use of biological and biochemical parameters to assess the ecological status of environmental samples provides accurate information about it. As reported by several authors [7–9], the biological parameters are largely modified by various environmental or anthropogenic factors and could be a potential indicator of ecological stress [10]. Microorganisms could play the role of environment quality bioindicators due to their quick reaction to adverse changes. In contrast to the physical and chemical properties which change very slowly, biological properties react quickly and they are sensitive even to small environmental fluctuations. In terms of ecotoxicological aspect, the research of microorganism populations in the test materials (soil, water, sediment, sewage) is essential as it provides the balance of the environment and in some restricted way counteracts of perturbing that balance [11].

Furthermore, microorganisms play an important role in many biological processes in order to circuit elements in the ecosystem and the decomposition of organic matter. It is important to assess the entire populations and the whole ecosystem because in this way, it is possible to obtain the most likely reflection of the natural environment conditions. The enzymatic activity of the microorganism populations is strictly correlated with its composition [12]. Changes in enzymatic activity could be the indicator of the changes occurring in the microorganism populations under a wide range of conditions.

Therefore, there is a need to carry out a research which aim is to characterize the microbial communities in the environmental samples. Considering the fact that the rapid community level physiological profiles (CLPP) [13] may be a helpful tool to understand the basic ecological aspects. According to studies of Garland and Mils [14] and Lehman et al. [15], the set of substrates for community characterization in environmental samples was developed.

The Biolog EcoPlate was created especially for community analysis and microbial ecology studies. The characteristics of the microbe community may be done by inoculating Biolog EcoPlate with a mixed culture of microorganisms or environment samples, for instance soil, water, wastewater, activated sludge, compost, and industrial waste. The Biolog EcoPlate System consists of 96-well microplates which every well is a coat lyophilize substrate (31 different carbon sources in three replications). The population of microorganisms gives a characteristic response pattern called a metabolic fingerprint [16].

Moreover, the definition of the community level physiological profiling has been demonstrated to be effective at temporal changes in microbial communities. Biological approach is crucial for the detection of pollution in the environment and the assessment of the sewage sludge toxicity [17]. The inhibitive effects of heavy metals and organic chemicals to microorganisms are key considerations in hazard management and control because microorganisms are ubiquitous in nature, and they are relevant for preserving the ecological balance [18].

During the past few years, there has been much effort to develop method and protocols which could guarantee the safety of industrial products as well as the protection and control of wastewater treatment plants [19]. Biolog EcoPlate approach could be helpful in estimating the effect of test factors on mixed bacterial communities in the aquatic environment. This method is a good detector of toxic compounds that could reduce or inhibit microbial activities. Applied EcoPlates in ecological research may be used as assay to detect and evaluate environmental changes.

In this context, the aim of the present work was to assess ecotoxicity of the dairy sewage sludge by using the Biolog EcoPlate approach. This research offers important data that supports the assessment quality of sewage sludge and their applications.

## Materials and Methods

### Experimental Section

The sludge used in the experiment was a waste-activated sludge (WAS) taken from a dairy wastewater treatment plant in Krasnystaw (Southeast Poland) which utilizes mechanical and biological methods at a flow rate of 200–2,000 m^3^. WAS is the final product of sewage treatment process of dairy. The type of sludge pollution is typical to dairy industry as follows: BOD_5_ 1,500–3,200 mg O_2_ dm^−3^, COD_5_ 250–4,000 mg O_2_ dm^−3^, suspension 300–1,000 mg dm^−3^, and pH 5–11. Raw sewage is fed into the tank gravity. It consists of holding a wastewater for a short period of time in a tank under quiescent conditions, allowing the heavier solids to settle, and removing the “clarified” effluent. Sedimentation for solid separation is a very common process operation and is routinely employed at the beginning and end of wastewater treatment operations. During the next step, flocks flow through the rapid sand filters under gravity, and the flocculated material (mineral and organic impurities) is trapped in the sand matrix. The pretreated wastewater is transferred into the aeration basin (tank), where it is mingled with the microorganisms. After spending time in aeration tank, wastewater overflows into a clarifier. Microbial fraction is settled by gravity and removed from the treated wastewater. A portion of the microbial biomass is recycled back to the aeration tank in order to maintain appropriate concentration of microorganism in the basin. At this stage, a sediment is also formed which is removed from the treatment system (WAS). After purification and disinfection, this part of sediment could be used as a fertilizer. The remaining sludge is pumped from the system for final disposal by chlorination, filtration, etc.

Because the biological properties of waste-activated sludge may shift with time and source [20, 21], the samples of waste were collected from treatment plants within a 5-month period (five samples of WAS were taken at monthly intervals). The collected sludge was immediately transferred to the lab and stored in a plastic box not beyond 24 h at 4 °C prior to use.

The aim of the study was to asses if the activity of microbial populations is changing in heavy metal-exposed sludge using the Biolog EcoPlate approach. Therefore, we compared the microbial metabolic profile in contaminated and uncontaminated waste-activated sludge. Waste-activated sludge as received from the treatment plant was rather low in heavy metals. Portions of the sludge were contaminated in laboratory by adding water-soluble chlorides or nitrates of the following heavy metals in order to reach the EU limits for heavy metals in sludge (option B): (A) Pb 3,000 mg kg^−1^ dry weight (dwt), Ni 1,000 mg kg^−1^ dwt, Cd 100 mg kg^−1^ dwt, Cu 4,000 mg kg^−1^ dwt, and Zn 10,000 mg kg^−1^ dwt and (B) Pb 1,500 mg kg^−1^ dwt, Ni 500 mg kg^−1^ dwt, Cd 50 mg kg^−1^ dwt, Cu 2,000 mg kg^−1^ dwt, and Zn 5,000 mg kg^−1^ dwt. The amount of heavy metals in option A is 100 % more than option B. Subsequently, the WAS were incubated for 7 days at 4 °C. The heavy metal effect on the sludge microbial community has been investigated by estimating influence of metals on community metabolic profile. Sludge characteristics are presented in Table 1.

### Community Level Physiological Profiling

The capability of sewage sludge microbial communities to utilize a variety of carbon sources was assessed by using Biolog EcoPlate [22]. Every plate had 96 wells containing 31 different carbon sources plus a blank well, in three replications. The rate of utilization of the carbon sources was pointed by the reduction of tetrazolium violet redox dye, which changed from colorless to purple if added microorganisms utilize the substrate [23]. EcoPlate was prepared in the following way: 1 g of sewage sludge was suspended in 99-ml sterile peptone water and shaken for 20 min at 20 °C and then was incubated at 4 °C for 30 min [24]. Next, each well of the Biolog EcoPlate was inoculated by 120 μl of the prepared suspension and incubated at 25 °C. Absorbance at 590 nm was measured on Biolog Microstation after 24, 48, 72, 96, 120, and 144 of incubation hours. Optical density (OD_i_) value from each well was corrected by subtracting the control (blank well) values from each plate well. Optical density values obtained at 120 h of incubation represented the optima range of optical density readings, so 120 h of incubation results was used for the assessment of microbial functional diversity and statistical analyses. In addition, substrates were subdivided into five group substrates, carbohydrates, carboxylic and ketonic acids, amines and amides, amino acids, and polymers, according to Weber and Legge [25].

Microbial activity in each microplate was expressed as average well color development (AWCD). Substrate richness values (*R*) were calculated as the number of utilized substrates and evenness were calculated according to Zak et al. [26] (Table 2).

**Table 2:** Formulae for calculations

Index	Definition	Formulae	Definitions
Average well color development		AWCD = Σ OD_i_/31	pi = proportional color development of the well over total color development of all wells of a plate *H* = Shannon index of diversity *S* = number of wells with color development (substrate utilization richness)
Shannon diversity	Measure of richness	*H* = −Σpi(lnpi)	
Shannon evenness	Evenness calculated from Shannon index	*E* = *H*/lnS	

### Statistical Analysis

AWCD, richness (*R*), and Shannon evenness (*E*) indices were investigated by analysis of variance (ANOVA). The cluster analysis was used to evaluate the substrates which were the most utilized in each sample of sludge, especially after heavy metal contamination. The data were standardized by the average well color development in each microplate to remove inoculum density effects [27]. All statistical analyses were performed with Statistica, version 10.

## Results

### Microbial Community Substrate Utilization Profile (CLPP)

Biolog EcoPlates were used to evaluate qualitatively and quantitatively community level physiological profile of microbe community in WAS.

**Fig. 1:**
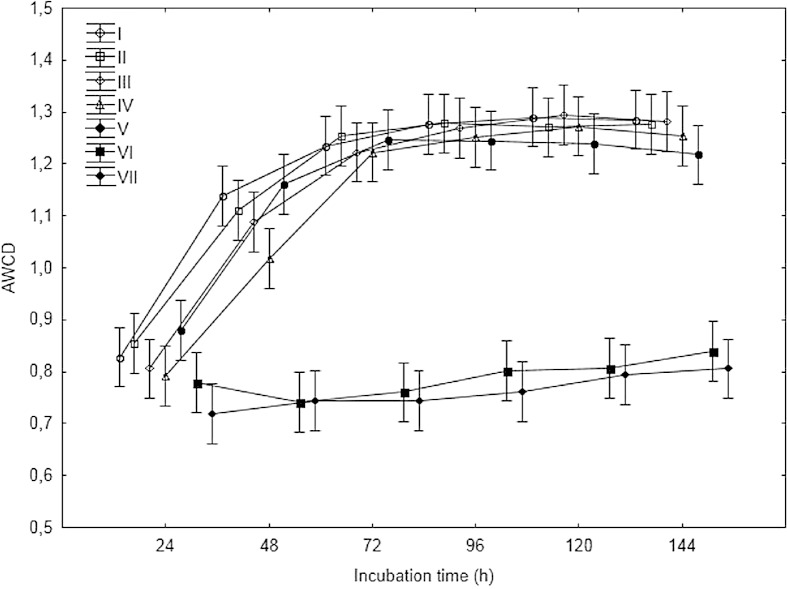
Average well color development (AWCD) of metabolized substrates in Biolog EcoPlates based on 144-h incubation (*n* = 3). Samples of waste-activated sludge (I–V) were taken in five terms, VI samples of WAS-contaminated heavy metal option A. and VII samples of WAS-contaminated heavy metal option B

Carbon substrate utilization, assessed via Biolog EcoPlates, showed that addition of heavy metals modified the metabolic potential of the WAS microbial community, and this effect was very intensive. The AWCD in the Biolog EcoPlates generally followed the same pattern with incubation time but varied for different WAS samples (Fig. 1).

In general, the WAS samples without heavy metals had the highest AWCD values. This implied that the microbial communities in WAS which was not contaminated by heavy metals had a stronger metabolic activity to utilize the carbon substrates. The order of AWCD values for different WAS samples was I > II > III > IV > V in the treatment of WAS without heavy metals and VI > VII in the treatment of WAS contaminated by heavy metals. There were no significant differences between the treatments of WAS without heavy metals (I–V) and the treatments WAS with heavy metals (VI–VII).

To further compare the catabolic diversity among different treatments, Shannon’s evenness (*E*) and substrate richness (*R*) in the incubation time of 120 h are shown in Table 3. For the WAS treatments without heavy metals (I–V), there were no significant changes in evenness and richness. However, significant differences in evenness and richness were found between the WAS (I–V) and contaminated treatments of WAS (VI–VII).

**Table 3:** Mean values of Shannon’s evenness (*E*) and richness of WAS (I–VII) based on 120-h incubation (means ± standard errors, *n* = 3)

Index	Treatments						
	I	II	III	IV	V	VI	VII
Shannon’s evenness (*E*)	0.997 ± 0.001	0.996 ± 0.0	0.997 ± 0.001	0.996 ± 0.001	0,997 ± 0.001	1.404 ± 0.2	1.168 ± 0.05
Richness (*R*)	31 ± 0.577	29 ± 0.577	30 ± 1	30 ± 1	30 ± 0.577	12 ± 3.605	15 ± 9.452

**Table 4:** Biolog EcoPlate carbon source guild groupings [24]

Well number	Carbon source	Compound group
A1	Water	–
B1	Pyruvic acid methyl ester	Carbohydrates
C1	Tween 40	Polymers
D1	Tween 80	Polymers
E1	α-Cyclodextrin	Polymers
F1	Glycogen	Polymers
G1	d-Cellobiose	Carbohydrates
H1	α-d-Lactose	Carbohydrates
A2	β-Methyl-d-glucoside	Carbohydrates
B2	d-Xylose	Carbohydrates
C2	i-Erythritol	Carbohydrates
D2	d-Mannitol	Carbohydrates
E2	*N*-Acetyl-d-glucosamine	Carbohydrates
F2	d-Glucosaminic acid	Carboxylic and ketonic acids
G2	Glucose-1-phosphate	Carbohydrates
H2	d,l-α-Glycerol phosphate	Carbohydrates
A3	d-Galactonic acid-γ-lactone	Carboxylic and ketonic acids
B3	d-Galacturonic acid	Carboxylic and ketonic acids
C3	2-Hydroxybenzoic acid	Carboxylic and ketonic acids
D3	4-Hydroxybenzoic acid	Carboxylic and ketonic acids
E3	γ-Hydroxybutyric acid	Carboxylic and ketonic acids
F3	Itaconic acid	Carboxylic and ketonic acids
G3	α-Ketobutyric acid	Carboxylic and ketonic acids
H3	d-Malic acid	Carboxylic and ketonic acids
A4	l-Arginine	Amino acids
B4	l-Asparagine	Amino acids
C4	l-Phenylalanine	Amino acids
D4	l-Serine	Amino acids
E4	l-Threonine	Amino acids
F4	Glycyl-l-glutamic acid	Amino acids
G4	Phenylethylamine	Amines/amides
H4	Putrescine	Amines/amides

**Fig. 2:**
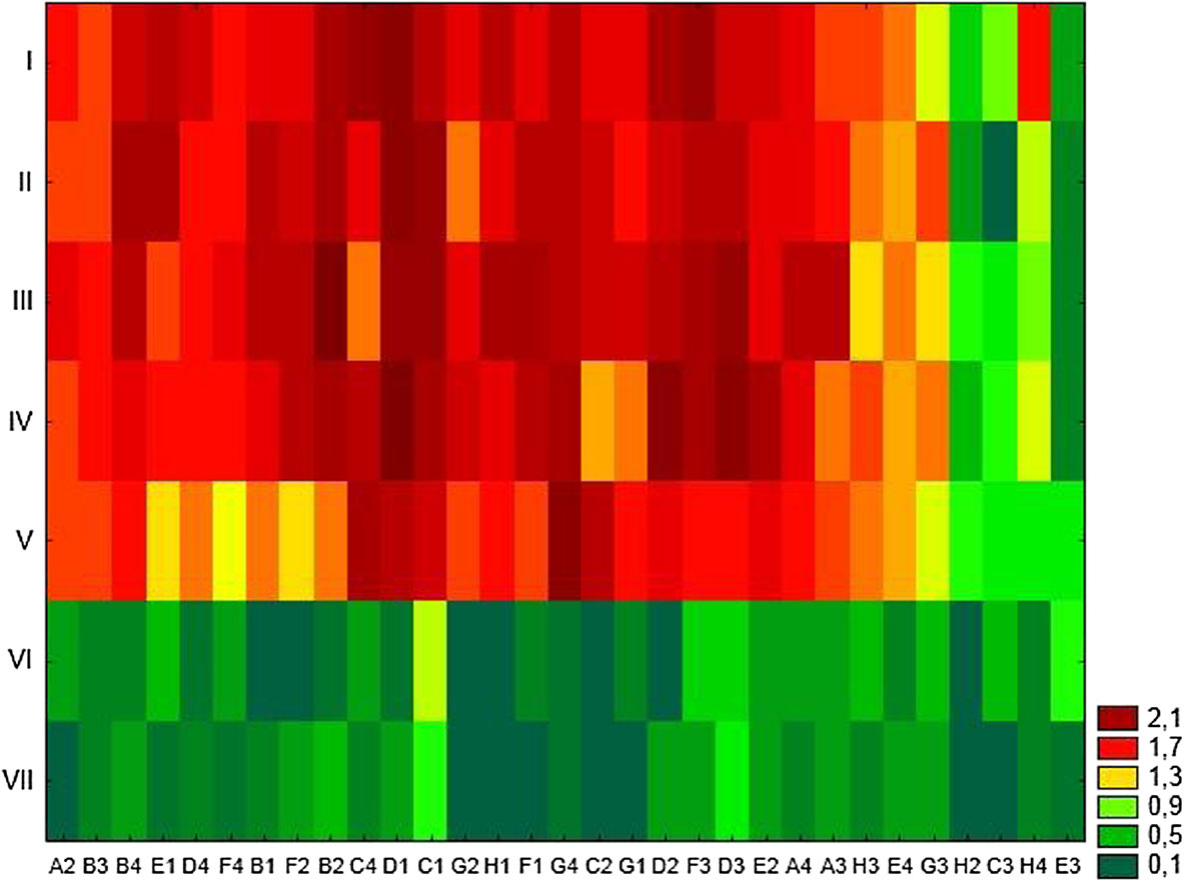
Results of cluster analysis of microorganism present in WAS samples depending on utilization carbon substrates in Biolog EcoPlate. Explanation: A2-E3 see Table 4

The wider and the highest metabolic activity was recorded from WAS I–V, which was able to metabolize 31 out of 31 substrates, while the WAS VI only 12 and WAS VII 19 substrates. The communities showed a metabolic diversification in the carbon source oxidation (Fig. 2).

The community in the WAS I–V was able to utilize all substrates; however, there were four substrates that were utilized at lower level than the vast majority of substrate (putrescine, γ-hydroxybutyric acid, 2-hydroxybenzoic acid, d,l-α-glycerol phosphate). The lowest utilization of all carbon sources was shown in the WAS VI–VII; in this treatment, only Tween 40 and 4-hydroxybenzoic acid were utilized at the highest level.

**Fig. 3:**
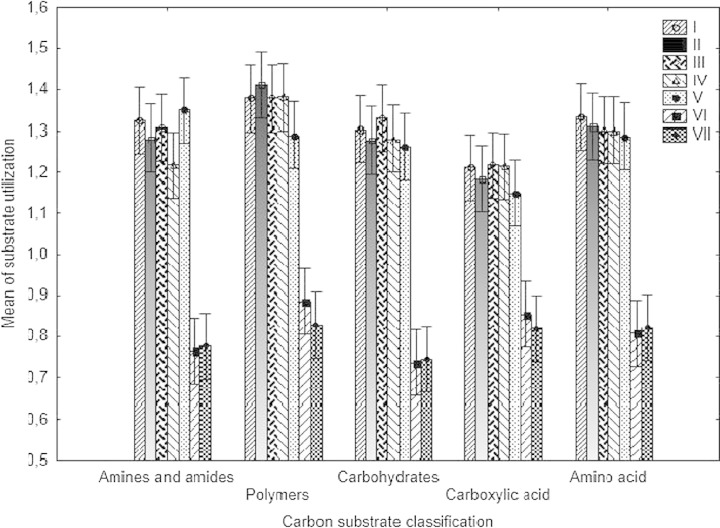
Mean of substrate utilization carbon substrates from different substrate groups by WAS microbes based on 120-h incubation (*n* = 3)

The community in the WAS VI–VII was unable to utilize glucose-1-phosphate, α-d-lactose, i-erythritol, and d,l-α-glycerol phosphate. The only substrate that both the communities did not oxidize was d,l-α-glycerol phosphate. Differences were not significant in the utilization of carbon source for the different guilds—amines and amides, polymers, carboxylic and acetic acid, carbohydrates, and amino acids (Fig. 3 Table 4).

## Discussion

Biological wastewater treatment is one of the most important parts of wastewater treatment plant. Activity of microorganisms participating with purification of wastewater may be a good indicator quality of waste-activated sludge. The Biolog plate is used for studying metabolic response of microbial communities from soil [28], estimating the influence of different land use [29], and for estimating compost maturity [30]. This technique is more and more frequently used for estimating the impact of stressing factor such as heavy metals [31] or hydrocarbon contamination [32], high salinity and high soil pH [33], or heating [34]. The Biolog method is more dedicated to compare research, e.g., for comparing functional diversity of microbial communities from contaminated and uncontaminated treatments rather than to characteristic microbial community [35, 36].

One of advantages of Biolog method is the inoculation of aquatic samples directly onto the plates, omission of the isolation stage. For this reason, the method is very interesting as a rapid and convenient tool for comparing functional diversity of whole microbe communities [37].

Described community level physiological profiles indicated differences between WAS functional diversity from the WAS without heavy metals and the WAS contaminated by metals. Heavy metals are known as harmful pollutants in environmental having negative effect on microorganism [38]. Therefore, the present study shows a strong influence of metal pollution on microbial activity. The obtained results indicate that metal pollution caused deterioration in the microbial activity and diversity of substrate utilization, which might be related to a reduction in catabolic functions [39].

Indices like AWCD, H, R, and S calculated based on results measured OD in microstation are very useful to describe activity and diversity of microorganism population. The community level physiological profile has been found to be a good indicator of reflecting changes of metabolic activity and/or potential functional versatility of microbial communities exposed to stress conditions, e.g., heavy metals [40]. The AWCD reflects the oxidative ability of microorganisms developed in Biolog, and it may be used as an indicator of microbial activity. The application of heavy metals to WAS significantly decreased AWCD in each treatment. It indicated that the activity of microorganism in WAS without heavy metals is higher than in WAS-contaminated metals. Additionally, calculation of richness index is also sensitive enough to evaluate microbial activity. High value of richness index indicates a high number of oxidized C substrates.

The activity of microorganism in waste-activated sludge could be a indicator of quality [39, 41]. Therefore, by using this method and parameters, it could be helpful to evaluate the quality of sewage sludge which may be used to fertilize soil [40].

## Conclusions

Due to contamination of sewage sludge with potentially hundreds of different substances (chemical and biological), environmental quality assessment of sewage sludge is challenging and has to be achieved with mixed methods: chemical and biological. Ecotoxicity assessment assures essential information about safety use of these materials. Biolog Ecoplates could be used as a test for the assessment of quality, for example when waste-activated sludge is targeted to agricultural or landscaping applications. It is important to use the test which is not only effective but also inexpensive, rapid, and easy to prepare. Assignment of metabolic profile is used for the identification and characteristic microorganisms. It is increasingly applied in research ecotoxicology to assess capabilities agricultural applications. This method is very responsive to pollution and toxic substances which could appear in waste materials.
